# In Silico Analyses of Autophagy-Related Genes in Rapeseed (*Brassica napus* L.) under Different Abiotic Stresses and in Various Tissues

**DOI:** 10.3390/plants9101393

**Published:** 2020-10-20

**Authors:** Elham Mehri Eshkiki, Zahra Hajiahmadi, Amin Abedi, Mojtaba Kordrostami, Cédric Jacquard

**Affiliations:** 1 Department of Agricultural Biotechnology, Payame Noor University (PNU), Tehran P.O. Box 19395-4697, Iran; elham.mehri66@gmail.com; 2 Department of Biotechnology, Faculty of Agricultural Sciences, University of Guilan, Rasht P.O. Box 41635-1314, Iran; z.hajiahmadi1366@gmail.com (Z.H.); abedi.amin@yahoo.com (A.A.); 3 Nuclear Agriculture Research School, Nuclear Science and Technology Research Institute (NSTRI), Karaj P.O. Box 31485498, Iran; kordrostami009@gmail.com; 4 Resistance Induction and Bioprotection of Plants Unit (RIBP)—EA4707, SFR Condorcet FR CNRS 3417, University of Reims Champagne-Ardenne, Moulin de la Housse, CEDEX 2, BP 1039, 51687 Reims, France

**Keywords:** codon usage bias, duplication, expression pattern, gene family, gene ontology

## Abstract

The autophagy-related genes (ATGs) play important roles in plant growth and response to environmental stresses. *Brassica napus* (*B. napus*) is among the most important oilseed crops, but *ATGs* are largely unknown in this species. Therefore, a genome-wide analysis of the *B. napus ATG* gene family (*BnATGs*) was performed. One hundred and twenty-seven ATGs were determined due to the *B. napus* genome, which belongs to 20 main groups. Segmental duplication occurred more than the tandem duplication in *BnATGs*. Ka/Ks for the most duplicated pair genes were less than one, which indicated that the negative selection occurred to maintain their function during the evolution of *B. napus* plants. Based on the results, *BnATGs* are involved in various developmental processes and respond to biotic and abiotic stresses. One hundred and seven miRNA molecules are involved in the post-transcriptional regulation of 41 *BnATGs*. In general, 127 simple sequence repeat marker (SSR) loci were also detected in *BnATGs*. Based on the RNA-seq data, the highest expression in root and silique was related to *BnVTI12e*, while in shoot and seed, it was *BnATG8p*. The expression patterns of the most *BnATGs* were significantly up-regulated or down-regulated responding to dehydration, salinity, abscisic acid, and cold. This research provides information that can detect candidate genes for genetic manipulation in *B. napus*.

## 1. Introduction

Autophagy consists of transferring the cargo into vacuole in plants, lysosomes, and animals and subsequently, decomposition [1]. In general, there are two types of autophagic processes, including macroautophagy and microautophagy [2]. Macroautophagy is a basic route for the decomposition of substrates using special double-membrane vesicles, called autophagosomes, to trap the materials [3]. Microphagy is a non-selective decomposition process in which the substances are directly decomposed by the lysosomes in animals or vacuoles in plants [4]. More than 36 types of autophagy-related genes (ATG) were already characterized, more than half of them encode core autophagy proteins and are conserved in the most studying organisms, including plants [5]. The most *ATGs* are involved in decomposition processes, but they also contributed to biosynthesis processes. In plants, there research about the role of these genes in growth, development, and responding to environmental stresses [6,7,8,9]. The *ATG* gene family is composed of several subfamilies in which *ATG4* and *ATG8* subfamilies have generally more members than other subfamilies in yeast, animals, and plants [10,11]. The plants are constantly exposed to environmental changes, which can be considered as biotic and abiotic stressors when they are too strong. Many molecular and physiological mechanisms are involved in plants’ tolerance to environmental stresses. The abiotic stresses include cold, heat, drought, salinity, nutrient deficiencies, heavy metal toxicity, and oxidative stress. Many parts of the world encounter the drought and salinity conditions limiting the crop yields. Abiotic stresses can increase the level of reactive oxidative species (ROS) in plants, which can be reduced by autophagy [12]. It is known that *ATG18a*, *ATG10b*, and *ATG8* are involved in responding to the salinity and drought stresses in *Arabidopsis*, rice, and wheat, respectively [13,14,15]. Likewise, the autophagosome formation was immediately induced under salinity conditions in *atg2* and *atg7 Arabidopsis* mutants, which displayed a hypersensitive phenotype [9]. The overexpression of many-core *ATGs* improves plant growth under abiotic stresses [16,17]. It has been reported that the destruction of *ATG* in *Arabidopsis* resulted in the accelerated leaf senescence [18]. Under normal conditions, the basal level of autophagy requires to maintain cellular homeostasis [19]. Although *ATG* mutants in the model plants such as *Arabidopsis thaliana*, *Oryza sativa*, and *Zea mays* can complete their life cycle, they have impaired in growth or stress response compared to wild-type plants [20]. It has been reported that *ATG* genes, in addition to playing a role in plant stress response, are involved in other plant biological processes such as nutrient metabolism, nutrient recycling, lipid metabolism, root development, aging, reproductive development, and crop yield [21,22]. Surveying the expression pattern of *Arabidopsis ATGs* in tissues and developmental stages indicated the specific and different functions of these genes in multiple tissues and cells [23]. For instance, *ATG8* is involved in developing the endosperm in maize and its expression increases from 18 to 30 days after pollination [24]. During the leaf senescence, the expression of 15 *Arabidopsis ATGs* is induced [25]. Likewise, during the seed development of *Arabidopsis*, all *ATGs* are already expressed in siliques [26]. *ATG* genes also play an important role in productivity and yield. For instance, rice *ATG7* knockout line caused the abnormal development of anther, spikelet sterility, decreased nitrogen efficiency, premature senescence leaf, and finally reduced the yield [27,28]. Autophagy plays an important role in the plant’s response to nutrient starvation, and the expression of *ATGs* is induced under carbon or nitrogen starvation. Maize plants with mutations in *ATG12* (*atg12*) have normal growth similar to wild-type plants under control conditions but, in nitrogen starvation conditions, stopped seedling growth, premature senescence leaf, and reduced seed production mutant plants have been observed [29].

Rapeseed (*Brassica napus* L.) is one of the most important oilseed crops in the world and is an allopolyploid due to the hybridization of *B. rapa* (2*n* = 2x = 20) and *B. oleracea* (2*n* = 2x = 18). This plant is used as one of the healthiest edible oils due to its high content of unsaturated fatty acids and proteins. *B. napus* oil contains omega-3, essential vitamins, and minerals [30]. *B. napus* are used as an excellent species for the genetic and molecular studies of development and adaptation to stresses such as heavy metals stress due to its outstanding features, including rapid growth, high biomass, accumulation of heavy metals in the stem without showing any symptoms. *B. napus* is also a good candidate for phytoremediation [31,32]. This plant is relatively cold and salinity tolerant. However, spring frost can cause serious damage and kill *B. napus* seedlings, followed by a severe reduction in yield [33]. Identifying the abiotic stress-responsive genes in *B. napus* can increase our knowledge to improve commercial *B. napus* cultivars and increase its tolerance to abiotic stresses such as cold and salinity. Although the genome of many plants is known, *ATGs* were studied in a few plants, including *Arabidopsis* [34], *O. sativa* [35], *Nicotiana tabacum* [36], *Z. mays* [29], *Capsicum annuum* [37], *Setaria italica* [38], *Musa acuminate* [39], *Vitis vinifera* [12], *Citrus sinensis* [40], and *Ricinus communis* [41]. Further investigations are necessary to characterize these genes in the various plant species to increase our knowledge about *ATGs* in plants. Therefore, in the present study, identification, evolutionary relationship, duplication, and selection pressure, exon–intron structure, promoter analysis, transcript-targeted miRNA, and simple sequence repeat markers (SSRs) prediction, RNA-seq data analysis, codon usage, and gene ontology of the *BnATGs* genes were investigated.

## 2. Results

### 2.1. The Identification of B. napus ATG Genes

To identify the *ATG* genes in the *B. napus* genome, the BLASTP program was applied using the query sequence of *Arabidopsis* [42], *C. Sinensis* [40], *V. vinifera* [12], and *O. sativa* [35] ATG proteins against the *B. napus* genome database [43]. One hundred and twenty-seven genes were determined after omitting the sequences without ATG domains (Supplementary Table S1). All *BnATGs* were grouped due to their specific domains and numbered based on the chromosomal locus except the *ATG2* subfamily, which was not identified. All identified *BnATGs* were encoded by gene families, including 26 members for *BnATG8*, 24 members for *BnATG18*, 11 members for *BnATG1*, eight members for each of the *BnATG20* and *BnVTI12*, six members for the *BnATG11*, five members for each BnATG4, *BnATG14*, and *BnATG101*, four members for each of the *BnATG3*, *BnATG5*, *BnATG6*, and *BnATG13*, three members for each of the *BnATG9*, *BnATG12*, and *BnTOR*, and two members for each *BnATG7*, *BnATG10*, *BnATG16*, *BnVPS15*, and *BnVPS34*. The physicochemical analysis of *BnATGs* was carried out using the ProtParam tool. The length of protein sequences encoded by these 127 *BnATGs* ranged from 93 amino acids of BnATG12b to 2478 amino acids of BnTORc. The molecular weight (MW) of BnATG proteins ranged from 10.29 to 278.5 kDa with the isoelectric points (pI) varying from 4.63 to 9.74 (Supplementary Table S1). Due to the prediction of subcellular localization, BnATGs have activity in nucleus, cytoplasm, chloroplast, mitochondria, plasma membrane, and extracellular space (Supplementary Table S1). The diversity in length and location of BnATG proteins indicates their diverse functions in various cellular processes.

### 2.2. The Phylogenetic Analysis of B. napus ATG Gene Family

**Figure 1:**
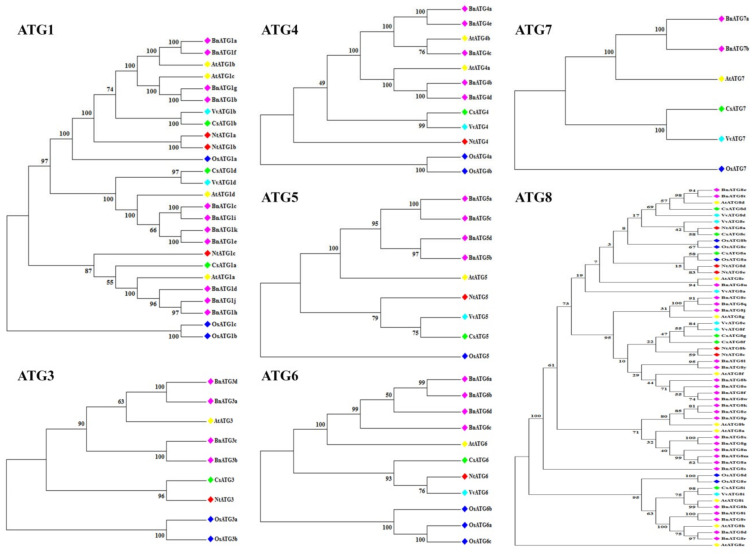
Phylogenetic relationships of BnATG1-BnATG8 proteins from *Brassica napus* (Bn), *Arabidopsis thaliana* (At), *Citrus sinensis* (Cs), *Oryza sativa* (Os), *Vitis vinifera* (Vv), and *Nicotiana tabacum* (Nt). The tree was constructed using MEGA 7 by the neighbor-joining (NJ) method with 1000 bootstraps. The names with pink rhombus are *B. napus* ATGs.

**Figure 2:**
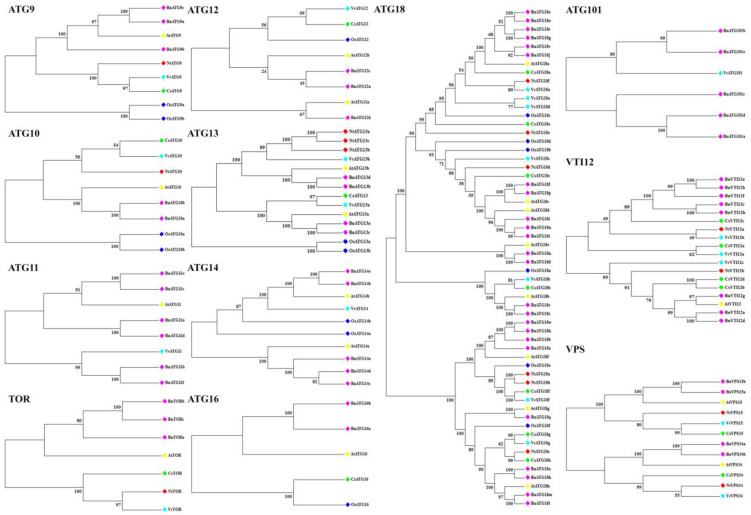
Phylogenetic relationships of BnATG9-BnATG114, BnATG16, BnATG18, BnVTI12, BnTOR, BnVPs15, BnVPS334, and BnVTI12 from *Brassica napus* (Bn), *Arabidopsis thaliana* (At), *Citrus sinensis* (Cs), *Oryza sativa* (Os), *Vitis vinifera* (Vv), and *Nicotiana tabacum* (Nt). The tree was constructed using MEGA 7 by the neighbor-joining (NJ) method with 1000 bootstraps. The names with pink rhombus are *B. napus* ATGs.

A neighbor-joining phylogenetic tree was constructed to assess the relationship of *B. napus* ATG proteins. The results indicated that each ATGs had high similarity to their counterparts in *Arabidopsis thaliana* (At), *Citrus sinensis* (Cs), *Oryza sativa* (Os), *Vitis vinifera* (Vv), and *Nicotiana tabacum* (Nt) (Figure 1 and Figure 2). Almost in all ATGs, OsATGs were clustered separating from the dicot clades. All BnATGs were clustered in different branches due to the existence of multiple isoforms except BnATG6–7, BnATG10, BnATG16, and BnTOR. Therefore, it can be suggested that they may have different functions in the same subfamily.

### 2.3. Gene Location, Duplication, and Selection Pressure of BnATGs

**Figure 3:**
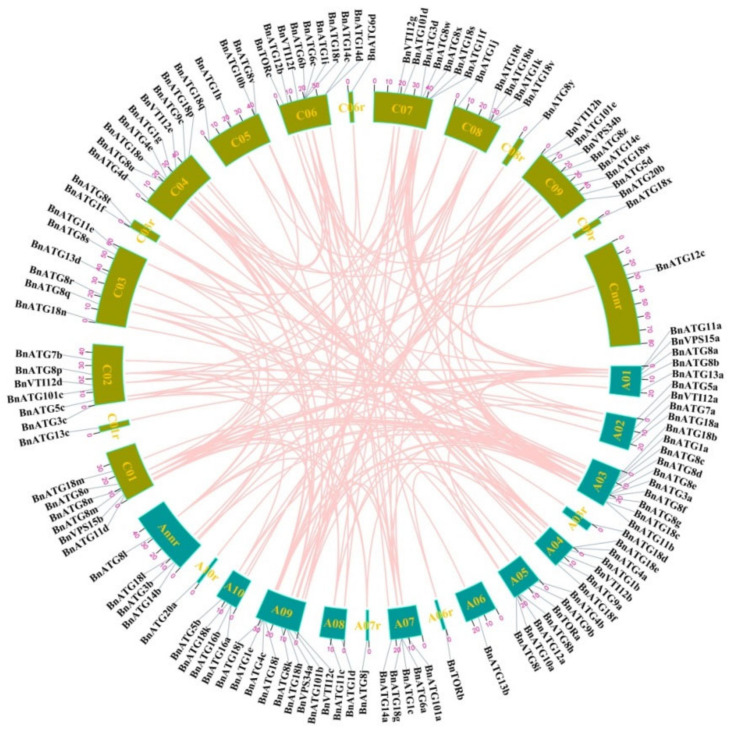
Chromosomal locations of *B. napus* autophagy-related genes. The location of genes on chromosomes and the duplication relationship between them were presented using TBtools. Chromosomes are represented by colored boxes. Pink curves connecting the genes indicate duplications.

Based on Figure 3, the chromosomal distribution of 127 *BnATGs* was uneven. Chromosome (Chr) A03, A09, C05, and C09 contained the highest number of *BnATGs*, whereas on ChA06, A6_random, A7_random, A10_random, C01_random, C06_random, C08_random, and C09_random, only one gene has been determined related to *BnATG13b*, *BnTORb*, *BnATG8j*, *BnATG20d*, *BnATG13c*, *BnATG6d*, *BnATG8y*, and *BnATG18x*, respectively. Based on gene duplication analysis, segmental duplication has occurred more than tandem duplication in the *B. napus ATG* gene family (Supplementary Table S2). Only five pairs of *BnATG* genes (*BnATG6b/BnATG6c* (ChC06)*, BnATG8n/BnATG8m* (ChC01)*, BnATG14c/BnATG14d* (ChC06)*, BnATG16a/BnATG16b* (ChA01), and *BnATG18u/BnATG18t* (ChC08)) revealed tandem repeats; thus, the role of segmental duplication in the expansion of the *BnATG* family is more important than tandem duplication. The Ka/Ks ratio of 165 paired genes was measured to show the selection pressure among duplicated genes (Supplementary Table S2). Ka/Ks was <1 for the most of paired genes, suggesting a negative selection to maintain their function during the evolution of *B. napus* plants. For three duplicated pairs (*BnATG16a/16d, BnATG*8m/*BnATG8a*, and *BnATG18g/BnATG18r*), the Ka/Ks ratio was >1, indicating a positive selection, leading to their different functions due to these mutations during the evolutionary process.

### 2.4. The Exon–Intron Structure and Conserved Motifs of B. napus ATGs

**Figure 4:**
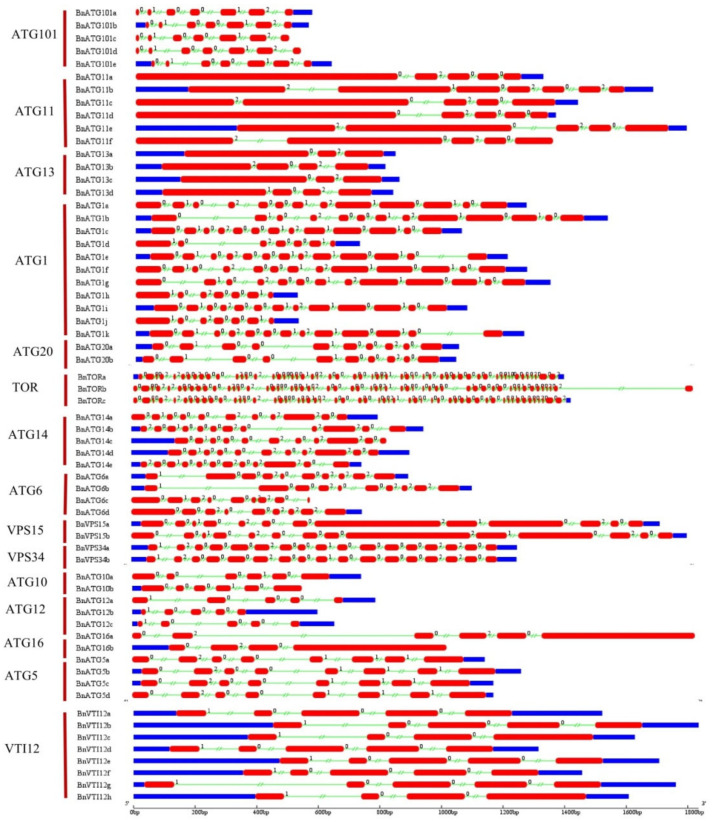
The exon–intron structure of *ATG1, ATG5–6, ATG10–14, ATG16*, and *ATG18* in *B. napus*. Exons and introns were represented by red boxes and green lines, respectively. The exon–intron structure of the *BnATGs* was determined using a gene structure display server (GSDS).

**Figure 5:**
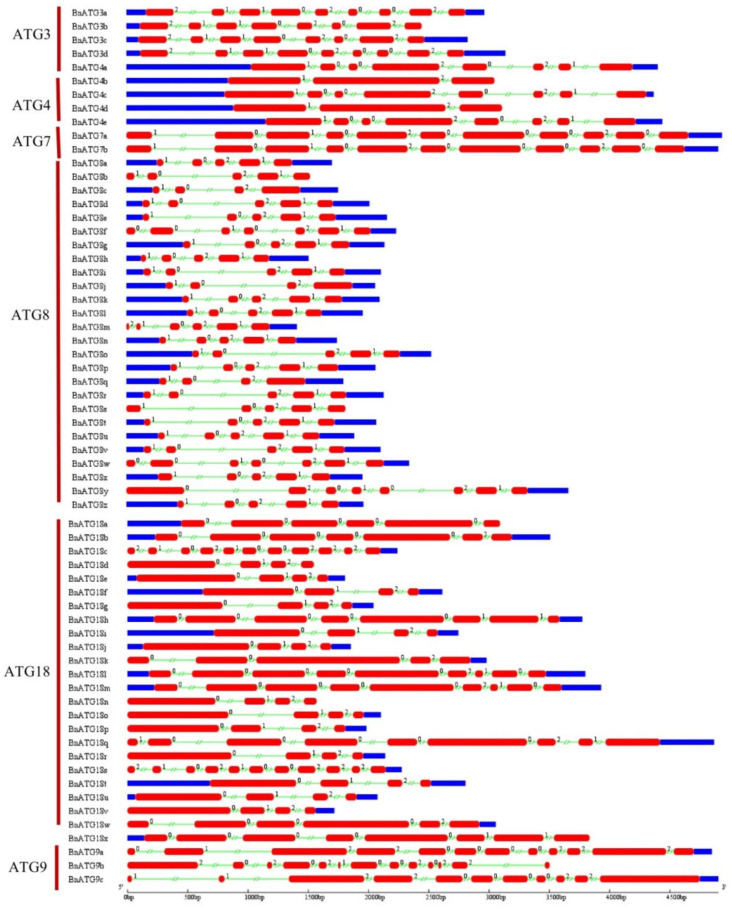
The exon–intron structure of *ATG3–4, ATG7–9, VPS15*, *VPS34*, *VTI12*, and *TOR* in *B. napus*. Exons and introns were represented by red boxes and green lines, respectively. The exon–intron structure of the *BnATGs* was determined using a gene structure display server (GSDS).

**Figure 6:**
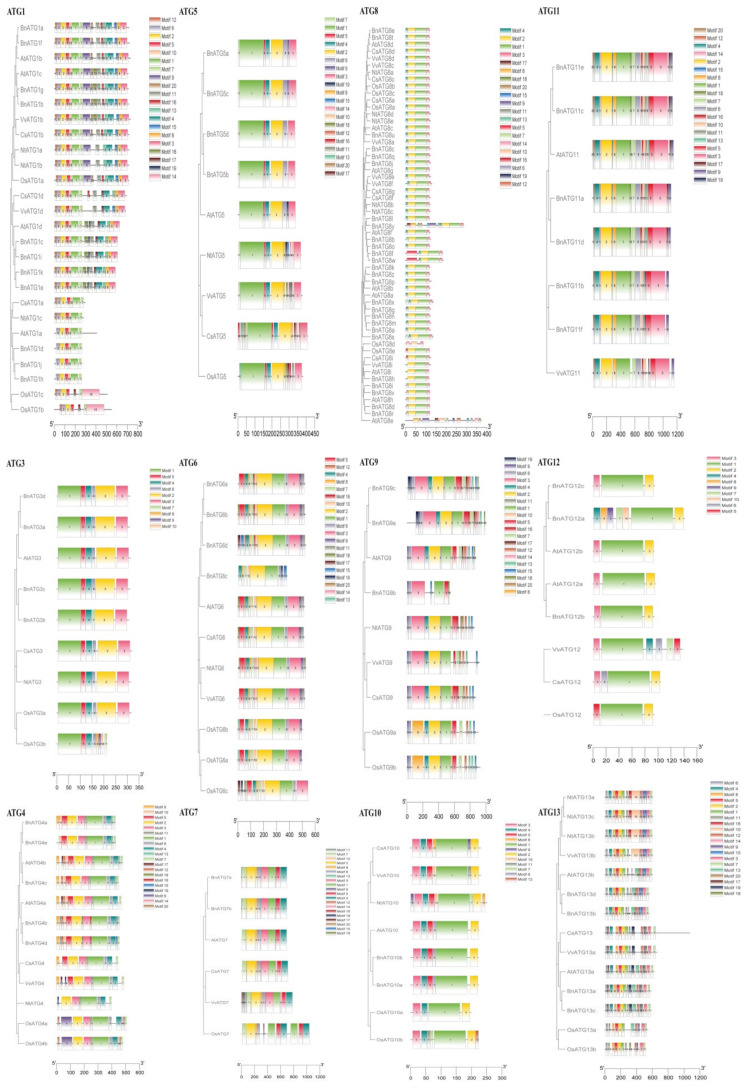
The conserved motifs of the *BnATG1–13*. Motifs were detected using the Multiple Em for Motif Elicitation (MEME) online tool. Different motifs are presented in different colors.

**Figure 7:**
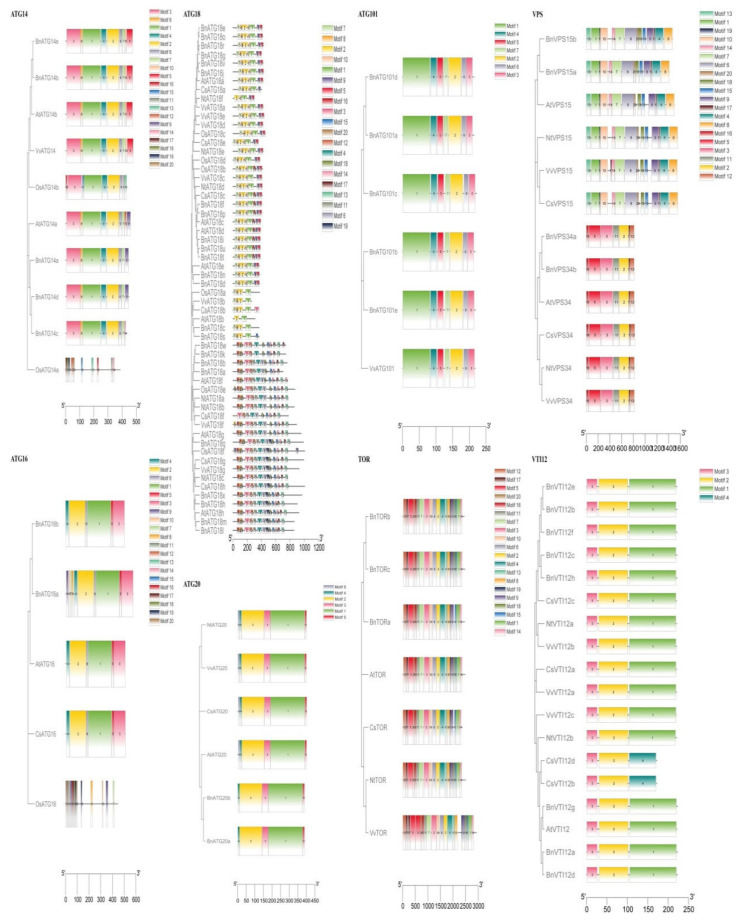
The conserved motifs of the *BnATG14–18, BnATG101*, *BnTOR*, *BnVPS*, and *BnVTI12*. Motifs were detected using the MEME online tool. Different motifs are presented in different colors.

Considering the exon–intron structure analysis of *BnATG*, two to 56 introns exhibited a high structural diversity among the *ATG* subfamilies (Figure 4 and Figure 5). *BnTORb* exhibited the longest intron. For the *B. napus ATG* gene family, three intron splicing phases were observed, including zero, one, and two, which resulted from splicing after the codon’s third, first, and second nucleotide, respectively [44]. Most of *BnATGs* had all three splicing phases except *BnATG10*, *BnATG12*, *BnVTI12*, and *BnATG16*. *BnATGs* had three to 17 exons, whereas three genes named *BnTORa–c* had 55 and 56 exons. All of *BnATGs* contained introns, but some had no untranslated region, including *BnATG6c*, *BnATG8b*, *BnATG9b*, *BnATG11f, BnATG16a*, *BnATG18d*, *BnATG18n*, *BnATG101c-d*, and *BnTORb*. The number of exons was similar in each ATG group. For instance, the *BnATG5*, *BnATG10*, *BnATG12*, and *BnATG20* revealed 8, 6, 5, and 10 exons, respectively. The diversity in the number of exons in some subfamilies indicated selective pressure to achieve different functions during plant evolution [45]. The similar exon–intron structure among A and C homologous copies showed that the homologous genes maintained their function during *B. napus* evolution. The intron splicing phases for each group were similar. *BnATG1*, *BnATG3, BnATG4* (except *BnATG4b* and *BnATG4d* with splicing phases zero and two), *BnATG5*, *BnATG6* (except *BnATG6d* with splicing phases zero and two), *BnATG*7, *BnATG8, BnATG9, BnATG14, BnATG15, BnATG34, BnTOR*, and *BnATG101* subfamilies revealed all three intron splicing phases. Likewise, in the *BnATG18* subfamily, all three splicing phases were observed except *BnATG18*a, *BnATG18b, BnATG18k, and BnATG18w* genes with splicing phases zero and two, *BnATG18b* and *BnATG18x* with splicing phases zero and one, and *BnATG18a* with only splicing phase zero. *BnATG11* (except *BnATG11b* with all three intron splicing phases) and *BnATG*16 subfamilies demonstrated splicing phases zero and two whereas *BnATG10, BnATG20* (except *BnATG20b* with all three intron splicing phases), and *BnVTI12* subfamilies showed splicing phases zero and one. To detect conserving motifs in BnATG proteins, Multiple Em for Motif Elicitation (MEME) was used (Figure 6 and Figure 7). Twenty motifs were nearly identified in almost all subfamilies, but the lowest number of motifs was observed in the BnVIT12 subfamily with four motifs, followed by ATG20 subfamily with six motifs. The main BnATGs conserved domains were Pkinase_Tyr, Autophagy_act_C, ATG, APG, WD40, BCAS3, Vps, PX, Peptidase_C54, PI3_PI4_kinase, and V-SNARE (Supplementary Table S3).

### 2.5. The Gene Ontology Annotations and Cis-Regulatory Elements of BnATGs

**Figure 8:**
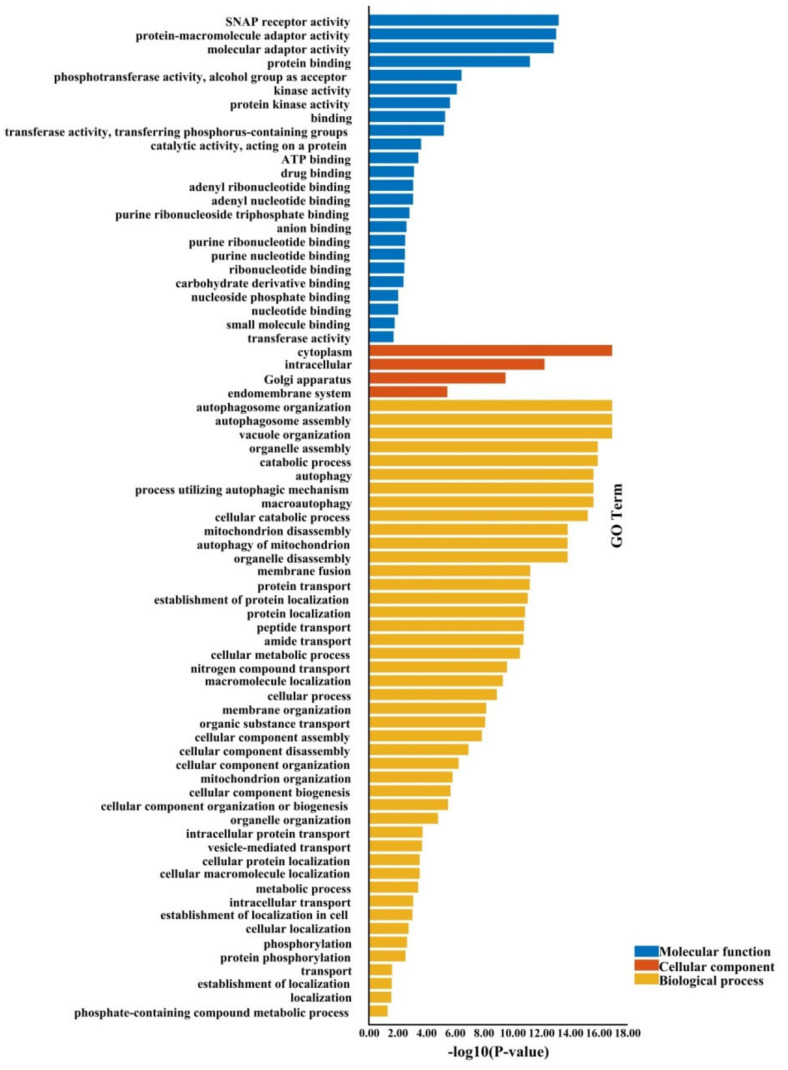
Gene ontology (GO) annotations of *ATG* genes in *B. napus*. GO analysis performed using TBtools.

The gene ontology (GO) annotations of 127 BnATGs proteins were determined using TBtools [46] due to GO terms (Figure 8, Supplementary Table S4). Considering Figure 8, BnATGs are involved in the various biological processes, molecular functions, and cellular components. As excepted, most of BnATGs are predicted to the function in autophagy-related processes including autophagosome organization (13 BnATGs), autophagosome assembly (13), vacuole organization (13), organelle assembly (13), catabolic process (52), autophagy (52), a process utilizing autophagic mechanism (52), macroautophagy (13), and cellular catabolic process (52). Moreover, the cellular component prediction indicated that rapeseed ATGs were primarily localized in the intracellular (45), which was followed by cytoplasm (43). BnATGs in the molecular function category were mainly involved in protein binding (39), soluble N-ethylmaleimide-sensitive factor attachment protein (SNAP) receptor activity (8), protein-macromolecule adaptor activity (8), and macromolecule adaptor activity (8).

To better evaluate the biological function of *BnATGs*, 1.5 Kb upstream of their transcriptional start codon (ATG) was examined using the PlantCare database to find cis-acting elements (Supplementary Table S5). A total of 91 cis-elements were found in the *B. napus ATG* gene family, which can regulate gene expression responding to five groups, including regulatory elements, light, developmental stages, phytohormones, and environmental stresses. The highest frequency of cis-elements was related to Myb (87.40%), followed by MYC (82.67%), ARE (76.37%), and G-box (70.07%). The lowest frequency of cis-acting regulatory elements was also related to re2f-1 (only in *BnVPS15b*), NON (only in *BnVPS15a*), L-box (only in *BnATG5d*), HD-Zip3 (only in *BnATG1h*), GATT-motif (only in *BnATG14a*), CAG-motif (only in *BnATG1h*), Box II-like sequence (only in *BnATG5d*), AUXRE (only in *BnATG18p*), and 4cl-CMA1b (only in *BnATG18m*). Eight hundred and sixty-three stress-responsive elements were found in *BnATGs*, indicating that they might have a potential function to regulate the *B. napus* response to various stresses. Likewise, 41, 22, 5, and 3 cis-regulatory elements were found in *BnATGs* related to meristem, endosperm, shoot, and seed, respectively (Supplementary Table S5). Due to the promoter analysis results, the *BnATGs* could have a potential function in different processes, including *B. napus* growth, development, and response to various environmental stresses.

### 2.6. The Prediction of Simple Sequence Repeats (SSRs) in BnATGs

137 SSRs were detected in 73 out of 127 *BnATG* genes (Table 1). Most genes had a single SSR except *BnATG9a* (7 SSRs), *BnATG9c* (6 SSRs), *BnATG101c* (5 SSRs), *BnTORa* (5 SSRs), *BnATG1b* (4 SSRs), *BnATG6a* (3 SSRs), *BnATG11b* (3 SSRs), *BnATG18a* (3 SSRs), *BnTORb* (3 SSRs), *BnATG18l* (4 SSRs), *BnATG8l* (3 SSRs), *BnATG8q* (3 SSRs), *BnATG18x* (3 SSRs), *BnATG20b* (3 SSRs), *BnATG101d* (3 SSRs), *BnVTI12b* (3 SSRs), *BnATG1a* (2 SSRs), *BnATG1f* (2 SSRs), *BnATG1i* (2 SSRs), *BnATG5a* (2 SSRs), *BnATG8c* (2 SSRs), *BnATG8d* (2 SSRs), *BnATG8h* (2 SSRs), *BnATG8j* (2 SSRs), *BnATG8y* (2 SSRs), *BnATG11a* (2 SSRs), *BnATG13a* (2 SSRs), *BnATG13d* (2 SSRs), *BnATG18m* (2 SSRs), *BnATG20a* (2 SSRs), *BnATG101e* (2 SSRs), *BnTORc* (2 SSRs), *BnVPS15a* (2 SSRs), *BnVPS15b* (2 SSRs), and *BnVTI12f* (2 SSRs). The highest frequency was related to tri-nucleotide repeats (62 SSRs), which were followed by tetra-nucleotide repeats (40 SSRs), di-nucleotide repeats (21 SSRs), penta-nucleotide repeats (11 SSRs), and hexa-nucleotide repeats (3 SSRs).

### 2.7. BnATG-Targeted miRNAs Prediction

**Figure 9:**
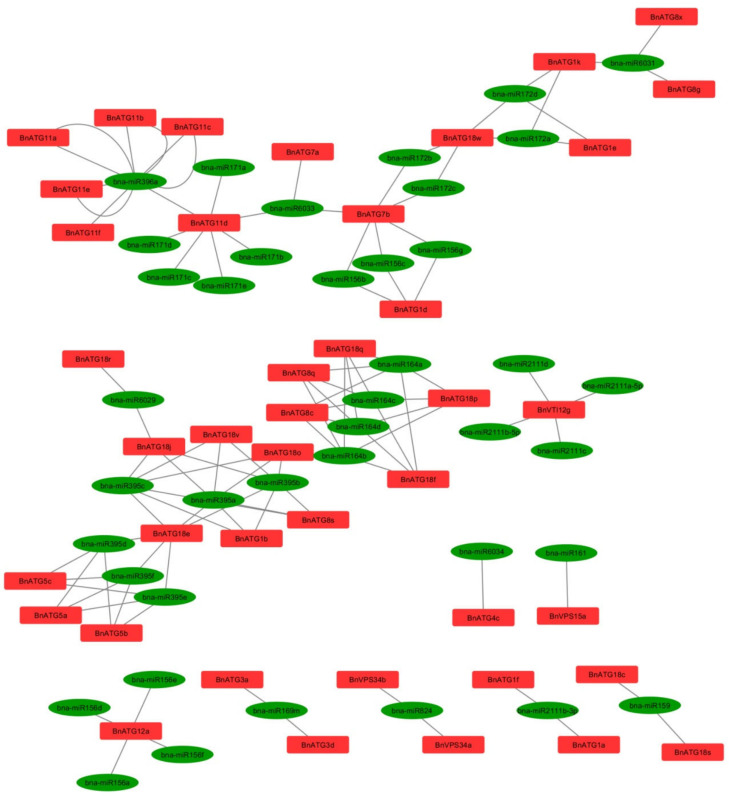
An miRNA-gene network based on interactions between miRNAs and *BnATGs*. Green ellipses represent miRNAs and red rectangles denote genes.

Due to the present results, 107 miRNAs for 41 *BnATGs* targets were identified (Figure 9 and Supplementary Table S6). miRNA’s relationship with their targets was not one by one, and many of them targeted the same gene. For example, *BnATG11d* transcript was co-targeted by seven miRNAs named bna-miR171a, bna-miR171b, bna-miR171c, bna-miR171d, bna-miR171e, and bna-miR6033. In contrast, one miRNA was predicted to target multiple transcripts, such as bna-miR395a, which can suppress the expression of *BnATG18e*, *BnATG18j*, *BnATG18o*, *BnATG18v*, *BnATG1b*, and *BnATG8s*.

### 2.8. The Expression Analysis of BnATGs at Various Developmental Stages

**Figure 10:**
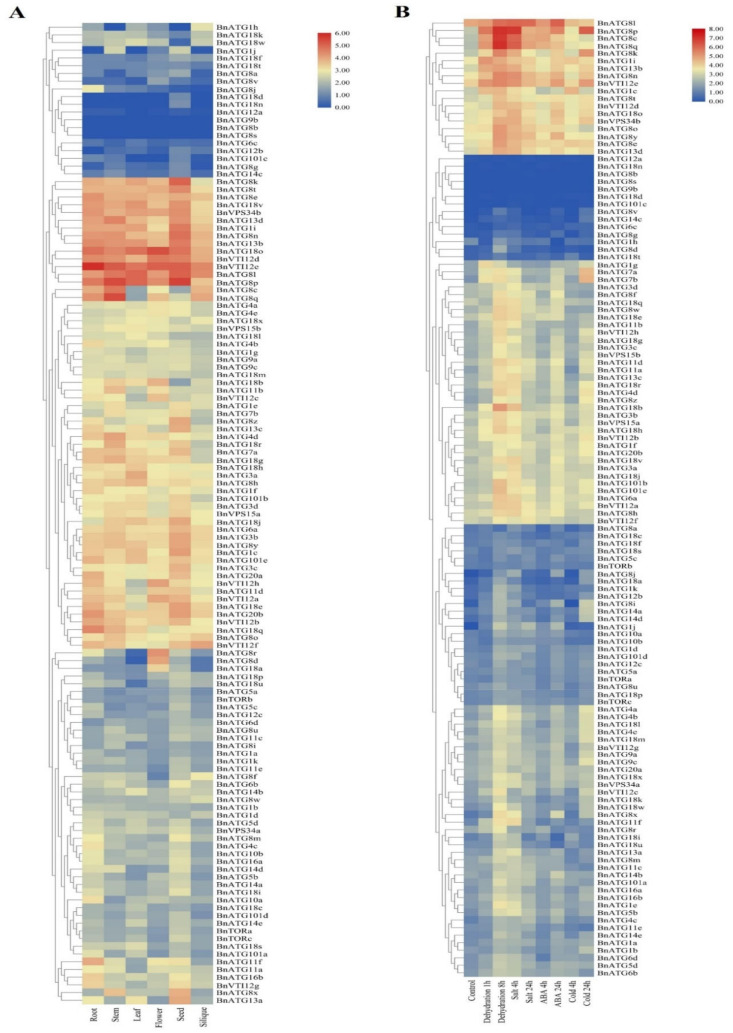
The expression pattern of *BnATG* genes (**A**) in different tissues, (**B**) under diverse abiotic stresses. The color boxes indicate expression values, the lowest (blue), medium (Pale goldenrod), and the highest (red). The heatmap was generated using log10 (TPM + 1) values.

The RNA-seq datasets of Zhang et al. [47] were analyzed to identify differentially expressed ATG genes in roots, stems, leaves, flowers, seeds, and siliques in *B. napus* under normal and stress conditions (Figure 10A). Most of the *BnATGs* were induced in the flowering development, while *BnATG8b, BnATG8s*, *BnATG18n, BnATGd, BnATGb*, and *BnATG101c* were repressed in this stage. Moreover, all *BnATGs* indicated low-to-high expression in all tissues of *B. napus*, whereas *BnATG1h* (in stem and seed), *BnATG1j* (in root and leaf)*, BnATG12b* (in root), *BnATG18d, BnATG18n* (in all tissues except seed), and *BnATG101c* (in leaf and flower) showed no obvious expression.

### 2.9. The Expression Profile of BnATGs under Abiotic Stresses

The RNA-seq datasets of Zhang et al. [47] for *B. napus* related to salt, cold, dehydration, and Abscisic acid (ABA) treatments were analyzed. The results (Figure 10B) indicated that the expression of autophagy genes depends on the stress type. For instance, *BnATG818b* expression was up-regulated under dehydration (after 8 h) and salt stresses (after 4 h), while it showed a moderate-to-low expression under other abiotic stresses. The highest gene expression response to dehydration (after 1 h) was related to *BnATG8p* (increased by 2.74 folds compared to control), while the highest expression in response to dehydration after 8 h was related to *BnATG8q*.

Furthermore, we found that *BnATG8p* expression was up-regulated in response to all abiotic stresses. Indeed, the expression of *BnATG8p* was 2.59, 1.70, 2.00, and 2.26 higher compared to control under dehydration (after 8 h), salinity (after 24 h), ABA (after 24 h), and cold (after 24 h) treatments, respectively. Under dehydration treatment, the expression of all *BnATGs* was increased, whereas the expression of *BnATG8a* and *BnATG12a* was reduced, and the expression of *BnATG8b, BnATG8s, BnATG9b*, and *BnATG101c* unchangeably remained. After 24 h under salinity stress, the expression of the majority of *BnATGs* was up-regulated except *BnATG8s, BnATG8v, BnATG9b, BnATG18n*, and *BnATG101c*, which exhibited a steady expression. Likewise, except *BnATG8b, BnATG8s*, and *BnATG9b* genes, which showed no expression, the level of *BnATGs* transcripts increased in response to ABA treatment. Down-regulated expression of *BnATG1h, BnATG8d, BnATG8v, BnATG10b, BnATG12a, BnATG18c, BnATG18i, BnATG18k, BnATG18s, BnATG18u*, and *BnVTI12f* genes was also found. The results of expression analysis were similar in the case of ABA and cold treatments except for *BnATG3d, BnATG6b, BnATG8f, BnATG8h, BnATG8l, BnATG8m, BnATG8o, BnATG8w, BnATG10a, BnATG11c, BnATG11e, BnATG18q, BnATG18t, BnATG18u, BnATG101a*, and *BnTORb* genes, which exhibited a down-regulation under cold stress and up-regulation under ABA treatment (Supplementary Table S7).

### 2.10. The Codon Usage Bias Analysis of BnATGs

The results of codon usage bias analysis of *BnATG* are presented in Supplementary Table S8. The GC value for studying genes was in the range of 0.38–0.55, and the GC3s value was calculated to be between 0.32 and 0.66. Due to the significant correlation between GC and GC3, the mutation is the main factor in the formation of codons. Indeed, if the correlation between these two parameters is significant, the mutation is the main factor in the formation of codons. If there had been no correlation between GC and GC3s, the main factor in codon formation would have been a natural selection [48]. codon adaptation index (CAI) is usually used to predict the expression levels of genes, which was in the range of 0.17–0.28 in BnATGs. The closeness of CAI to 1 implies a stronger codon preference and higher gene expression.

**Figure 11:**
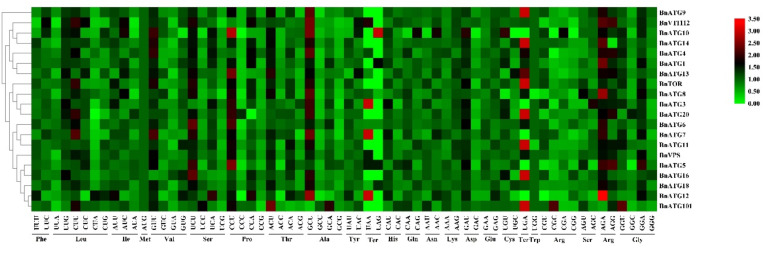
Heat map of relative synonymous codon usage analysis (RSCU) values of BnATGs. The color boxes indicate RSCU values, the lowest (green), and the highest (red) usage of codons. The heatmap was generated using TBtools.

A relative synonymous codon usage (RSCU) > 1 shows that the codons are applied more than other synonymous, an RSCU = 1 indicates no preference for codons, and if RSCU < 1, the codons are rarely used by genes [49]. There are 32 codons in *BnVPS34*; 31 codons in *BnATG1, BnATG9*, and *BnATG11*; 30 codons in *BnATG3, BnATG5, BnATG7, BnATG8*, and *BnATG14*; 29 codons in *BnVTI12*; 28 codons in *BnATG*16, *BnATG20, BnATG101*, and *BnTOR*; 27 codons in *BnATG4, BnATG6, BnATG10*, and *BnATG13*; and 26 codons in *BnATG12* with RSCU > 1, showing that these codons are the preferred codons for each gene subfamily. The higher RSCU values showed more frequent codons for each subfamily shown in red, while the lower RSCU value is indicated in green color (Figure 11). As shown in Figure 11, *BnATGs* were classified into three clusters based on the RSCU value, including cluster I (*BnATG9*), cluster II (*BnATG1, BnATG4, BnATG10, BnATG14, BnATG13*, and *BnVTI12*), and cluster III (*BnTOR, BnVPS, BnATG101, BnATG3, BnATG5, BnATG6, BnATG7, BnATG8, BnATG11, BnATG12, BnATG16, BnATG18*, and *BnATG20*). Each cluster showed a similar codon preference.

## 3. Discussion

In present study, a total of 20 core *ATGs* (*ATG1*, *ATG3–4*, *ATG6–14, ATG16, ATG18*, *ATG20*, *ATG101*, *VPS15*, *VPS34*, *TOR*, and *VTI12*) including 127 members of *ATG* gene family were identified in *B. napus*, which is considerably greater than the number of *ATG* genes in *Z. mays* (45 genes) [29], *Arabidopsis* (40 genes) [42], *S. italica* (37 genes) [38], *V. vinifera* (35 genes) [12], *C. sinensis* (35 genes) [40], *O. sativa* (33 genes) [35], *M. acuminate* (32 genes) [39], *N. tabacum* (30 genes) [36], and *C. annuum* (29 genes) [37]. The *ATG2* subfamily was not identified in the *B. napus*. Due to the number of identified *ATG*s in plants, it can be concluded that there is no significant relationship between genome size and the number of genes. For instance, foxtail millet and tobacco have 37 and 30 *ATG* genes, respectively, while the genome size of these two plants is 490 Mb and 4.5 Gb, respectively. The segmental and tandem duplications can affect the formation of gene families. Therefore, the observed difference in the number of identified *ATGs* may be related to the duplication during plant evolution. The identified *BnATGs* were categorized into ATG1/13 kinase complex, PI3K complex, ATG9/2/18 complex, ubiquitin-like ATG8, and PE conjugation pathway, ubiquitin-like ATG12, and ATG5 conjugation pathway, and SNARE groups (Supplementary Table S1). *BnATG8*, *BnATG18*, and *BnATG1* had multiple copies compared to other core *BnATGs* with 26, 24, and 11 members, respectively. The uneven distribution of *ATGs* has already been observed in rice [35], grapevine [12], and wheat [50]. The *BnATGs* were widely distributed in *B. napus* genome, which can be related to its different ancestors. Studying the gene duplication events is necessary to better understand the expansion of the gene family and the role of genes. In the present survey, both segmental (96.96%) and tandem (3.04%) duplications resulted in the generation of multiple copies of *ATG* genes in *B. napus.* The Ka/Ks ratios of the most duplicated *BnATGs* were <1 except for three duplicated gene pairs (*BnATG16a/16d, BnATG*8m/*BnATG8a*, and *BnATG18g/BnATG18r*) with Ka/Ks > 1 and two duplicated gene pairs (*BnATG1h*, BnATG1j, and *BnATG18u/*BnATG18t) with no Ka/Ks value due to the same sequence. It is well known that the alterations in the coding region of duplicated genes during the evolution resulted in different functions associated with amino acid substitution or divergence in exon–intron structure [51]. In general, the importance of the functional role of *ATGs* in *B. napus* was determined due to the strong purifying selection in the *BnATG* gene family.

Considering the phylogenetic tree, a close relation of BnATGs with their counterparts was observed, which related to their sequence conservation and similar function. All members of ATG subfamilies showed similar motif distribution, suggesting that the protein structure was conserved in each subfamily. The phylogenetic distribution of *B. napus* ATG proteins were associated with their motif compositions. BnATG1 subfamily had common motifs 1–2, 5–7, and 10. The difference among the motif compositions of BnATG1a/BnATG1f and BnATG1g/BnATG1b was only the existence of motif 18 in BnATG1g/BnATG1b cluster. Likewise, BnATG1c/BnATG1i/BnATG1k/BnATG1e cluster was separated from the two clusters mentioned above due to the lack of motifs 9 and 20 and the existence of motifs 13 and 17. The BnATG1d/BnATG1j/BnATG1h cluster was completely separated from other BnATG1s due to the lack of motifs 3–4, 8–9, and 11–20. BnATG5s, BnATG7s, BnATG11s, BnATG20s, BnATG101s, BnTORs, and BnVIT12s demonstrated the same motif contents for each subfamily, while the BnATG4b/BnATG4d cluster of BnATG4 subfamily had specific motifs 15, 16, and 18; thus, it clustered in a separated clade. The BnATG6 subfamily, BnATG6c was separated from other BnATGs due to the lack of common motifs 3, 5, 9, 11–12, and 16. BnATG8s were clustered into three clades due to their evolutionary relationships which are similar to their motif contents (Figure 1). Clade I contained motifs 1–4 except for BnATG8o/BnATG8w with motif 5 and BnATG8y with motifs 8–9, 15, 17–18, and 20. The motif contents of clade II were similar to clade I (motifs 1–4) except for BnATG8x and BnATG8s with motif 7. The clade III members contained motifs 1–3, and 6 except for BnATG8h with the lack of motif 6. BnATG9b had only motifs 1, 3, 5–6, 9, 15, and 18; thus, its cluster was separated from other BnATG9s. The difference between BnATG13d/BnATG13b cluster and BnATG13a/BnATG13c clusters was related to the existence of motifs 17–19 in the second cluster. The BnATG14e/BnATG14b cluster was also separated from the BnATG14a/BnATG14d/BnATG14c cluster due to the existence of motifs 5 and 16. BnATg18s were clustered into III groups. Common motifs of Clade I were 1, 2, 5, and 7–10, while clade II contained motifs 1, 2, and 7–8. Likewise, special motifs 3–4, 8, 12–18, and 20 were detected in clade III. BnVPS34 subfamily was completely separated from the BnVPS15 subfamily due to the different motif compositions. Exon–intron structure and splicing phase are important factors in the evolution of gene families [52]. Intron phase 0 and 1 showed the highest and high conservations, respectively, while intron phase 2 indicates the lowest conservation [53,54]. The rate of phases zero and one in all subfamilies including *BnATG1* (84.21%), *BnATG3* (60%), *BnATG4* (66.66), *BnATG5* (89.28%), *BnATG6* (55.88%), *BnATG7* (80%), *BnATG8* (74.31%), *BnATG9* (56.25%), *BnATG10* (100%), *BnATG12* (100%), *BnATG14* 67.3%), *BnATG16* (62.5%), *BnATG18* (76.06%), *BnATG20* (88.8%), *BnVTI12* (100%), *BnTOR* (85.54%), *BnATG101* (83.33%), *BnVPS15* (76%), and *BnVPS34* (75%) was higher than phase two except for *BnATG11* (50%) and *BnATG13* (50%), which ascertained the low diversity in the structure of these genes and high conservation in protein function of the *BnATG* family.

SSRs are short tandem repeats of a simple motif of 1–6 nucleotides, reported to play a significant role in controlling gene expression [55]. In the current paper, tri-nucleotide repeats (45.25%) were higher than other SSRs. The type of dominant SSRs is taxon-dependent, which varies in different plant species, and in general, the frequency of AT repeats is higher in dicot than monocots [56]. In future research, SSR polymorphisms in *BnATGs* may be suitable to select the genotypes with higher levels of resistance to abiotic stresses using marker-assisted selection techniques.

miRNAs are a group of 19–24 bp non-coding small RNAs, playing significant roles in plant growth and response to the environmental stresses through post-transcriptional changes [57]. Therefore, bioinformatics methods have helped predict the target of miRNAs in the shortest possible time. In *BnATG1*, *BnATG3*, *BnATG4*, *BnATG5*, *BnATG8*, *BnATG12*, *BnATG18*, *BnVPS15*, and *BnVTI12* subfamilies, six (*BnATG1a-b*, *BnATG1e-f*, and *BnATG1k*), two (*BnATG3a* and *BnATG3d*), one (*BnATG4c*), five (*BnATG5a-c*), five (*BnATG8c*, *BnATG8g*, *BnATG8q*, *BnATG8s*, and *BnATG8x*), one (*BnATG12a*), 11 (*BnATG18c-f, BnATG18j18o-s*, and *BnATG18v-w*), one (*BnVPS15a*), and one (*BnVTI12g*) transcripts were targeted by *B. napus* miRNAs, respectively. Although no target was found in *BnATG6*, *BnATG9*, *BnATG10*, *BnATG13*, *BnATG14*, *BnATG16*, *BnATG101*, and *BnTOR* subfamilies, all members of *BnATG11*, *BnATG7*, and *BnVPS34* were targeted by miRNAs. mir156 is essential to regulate plant transition time from a juvenile to an adult in the vegetative phase [58]. Therefore, it was shown that miR172 is involved in regulating flowering time [59,60]. miR159, miR169, and miR395 play a role in the regulation of seed germination, response to salinity stress, and sulfate starvation, respectively [61,62,63]. Likewise, miR61 and miR171 are down-regulated under salinity and up-regulated under drought conditions in *B. napus*, respectively [64]. miR164 and miR396 are involved in seed germination and response to abiotic stresses [65,66,67], and miR824 is progressively accumulated in response to heat exposure [68]. miR2111 is important in response to phosphorus deprivation, which is ascertained in *B. napus* [69]. Finally, miR6029 is necessary for fatty acid biosynthesis during *B. napus* seed development [70].

Considering promoter analysis, the highest number of stress-responsive elements was observed in *BnATG8r* with 12 of 20 stress-related cis-elements. The existence of regulatory elements associated with response to stresses, light, and hormones indicates that *BnATGs* are involved in the plant response to various stresses and biological processes. Likewise, GO annotation found that most *BnATGs* are included in the biological process, most of which are related to stress conditions response. The presence of different regulatory elements, involved in various biological processes, in the promoter of *BnATG* genes and post-transcriptional regulation of these genes by miRNAs show the complex and precise mechanism regulating the expression of these genes that leads to the various functions of *BnATGs*.

An analysis of the expression profile data published by Zhang et al. [47] indicated that the expression profile of genes helps to determine their function. The expression analysis indicated that most *BnATGs* are involved in *B. napus* development and response to abiotic stresses, which is in line with previous researches [9,40,42]. For instance, *BnATG*1c, *BnATG*1i, *BnATG3a-d, BnATG*6a, *BnATG7a, BnATG8e, BnATG8l, BnATG8n, BnATG8k, BnATG8t, BnATG8p*, *BnATG*8x, *BnATG8y, BnATG8z, BnATG13a-d, BnATG18e, BnATG18o, BnATG18v*, *BnATG20a-b, Bnvps34b, BnVTI12a-b*, and *BnVTI12d-f* revealed a high level of transcripts in seed, suggesting that they may be involved in the regulation of seed development. RNA-seq data analysis revealed that the *ATG* gene expression, in response to abiotic stresses, depends on the stress type and duration. These findings are inconsistent with those of Shangguan et al. [12]. For example, *BnATG18b* and *BnVTI12* genes were up-regulated by salt after 4 h of treatment, while they were repressed after 24 h under salinity condition. Considering the present study, the expression level of *BnATG8p* and *BnATG8q* was the highest among other *BnATGs* under dehydration conditions after 1 and 8 h, respectively. Likewise, the expression of *BnATG8p* and *BnATG8l* was the highest under salinity and ABA treatment. The transcript levels of *BnATG1c* and *BnATG8p* were also higher than other *BnATGs* in response to cold stress after 4 h and 24 h, respectively. These results indicated the potential key role of these genes in *B. napus* response to abiotic stresses, which can be used in future research to develop stress-resistant cultivars. The *BnATGs* exhibited different expression patterns, even in the same subfamily. For instance, in the *BnATG8* subfamily, *BnATG8l, BnATG8n, BnATG8p*, and *BnATG8q* were significantly up-regulated by dehydration, while no obvious changes in *BnATG8s* and *BnATG8v* expression was observed. In general, the expression of the *BnATG8* subfamily under all abiotic stresses was higher than other subfamilies, which is the following results obtained in *Arabidopsis* [38], foxtail millet [50], and wheat [38,50,71]. ATG8 is a ubiquitin-like protein conjugated to phosphatidylethanolamine (PE) catalyzed by ATG7, ATG3, and ATG12-ATG5 complex [72]. ATG8-PE complex is essential for autophagosome formation through the membrane connection and remodeling [73]. The expression of *ATG8* in wheat and *Arabidopsis* increases plant tolerance to osmotic stress [13,74]. The expression of *GmATG8c* in *Arabidopsis* and *SiATG8a* in rice also improved plant tolerance to nitrogen deficiency [38,75]. Likewise, *ATG8* showed a response to leaf senescence due to nitrate conditions in barley [76].

In this study, the factors involved in codon usage bias (CUB), including expression level, GC value, and mutation were investigated. CUB index varies among genes in each genome. Effective codon number (ENC) value was used to evaluate the effective codon number between 20 and 61.20 [77]. In this study, the ENC value was between 43.8 and 61, indicating that there are various synonymous codons among studying genes. Highly expressed genes have a higher codon preference with higher CAI and lower NC values. However, the genes with low expression have more types of rare codons; thus, they have a lower codon preference and a lower CAI but a higher NC. *BnATG8p, BnATG8q*, and *BnATG8l* genes, which had higher expression under different abiotic stresses, revealed almost higher CAI and relatively lower NC. Most of the *BnATGs* showed GC content < 0.5, indicating that *B. napus ATGs* have no perceptible preference for GC nucleotides. Only 12.59% of *BnATGs* revealed GC3s value > 0.5, indicating that codons with A/T end are preferred.

## 4. Materials and Methods

### 4.1. In Silico Identification of BnATG Genes

To determine the *BnATG* gene family in *B. napus*, related protein sequences in *Arabidopsis* [42], *O. sativa* [35], *C. sinensis* [40], *N. tabacum* [36], and *V. vinifera* [12] were obtained from Phytozome 12.1.6 database [78]. Then, the protein sequence of *ATG* genes related to the mentioned plants was used to identify *BnATG* genes in the BRAD database [43] using the BlastP algorithm (E.Value < 1e^−5^) [41]. The existence of ATGs domains in the obtained sequences was assessed using HMMscan databases [79]. Sequences without ATG domains were deleted, and the remaining genes were classified into different ATG groups based on the specific domain of each group. The genes were named by first adding Bn (*Brassica napus*), and then adding the name of the group and the English letters based on the chromosomal location of the genes. Molecular weight (M_W_), length, and theoretical isoelectric points (pI) of *B. napus* ATGs were calculated using the ProtParam tool of the ExPASY database [80]. To identify the cellular localization of proteins, subCELlular LOcalization (CELLO) has been applied [81].

### 4.2. Phylogenetic Analysis of B. napus ATG Gene Family

Full-length protein sequence alignment of *B. napus*, *Arabidopsis*, rice, sweet orange, tobacco, and the grapevine was handled using ClustalX 2.0.8 program to evaluate the evolutionary relationships of the *ATG* gene family. A phylogenetic tree of ATG proteins with 1000 bootstraps [82] constructed using MEGA 7 [83] by the neighbor-joining (NJ) method.

### 4.3. Chromosome Localization, Gene Duplication, and Selection Pressure

*ATGs* location on *B. napus* chromosomes was obtained from the *Brassica* database (BRAD) database [43]. Genes with a maximum interval of 10 genes on the same chromosome were regarded as tandem duplication [84]. Two criteria are considered to recognize segmental duplication, including more than 80% identification of the aligned region and more than 80% alignment coverage compared to the longer genes [85]. DnaSP ver. 5 software [86] was applied to compute the substitution rates of non-synonymous (Ka) per synonymous (Ks) of the duplicated genes to determine the type of selection pressure. The location of genes on chromosomes and the duplication relationship among them were presented using TBtools [46].

### 4.4. Exon–Intron Structure and Conserved Motifs

The gene structure of *BnATGs* was assessed using a gene structure display server (GSDS 2.0) [87]. This server evaluates the genomic DNA sequence of each gene based on its coding sequence and presents the exon, intron, 3′-UTR, 5′-UTR, and intron phase of the gene. Multiple Em for Motif Elicitation (MEME 5.0.5) was used to identify the conserved motifs of the *ATG* gene family [88]. A limit of 20 motifs, and minimum and maximum motifs length were 10 and 200, respectively.

### 4.5. Gene Ontology Annotations and Cis-Regulatory Element Identification

To investigate the functional role of *ATG*s in *B. napus* biology, gene ontology (GO) analysis was performed using TBtools [46]. Three levels of GO classification, including molecular functions, biological processes, and cellular components, were applied to present genes. An amount of 1.5 kb upstream of the initiation codon of *ATG* genes was retrieved from the Ensemble Plants database [89], and cis-acting regulatory elements were identified using PlantCare [90]. Only cis-elements with scores ≥ 6 were examined.

### 4.6. The Prediction of Simple Sequence Repeats (SSR) Markers and BnATG-Targeted miRNAs

SSR markers were detected in *BnATG* genes using the BatchPrimer3v1.0 server [91]. In the psRNATarget database, CD sequences of them were examined by considering default parameters to find *BnATG*-targeted miRNAs. The relationship between *BnATGs* and identified miRNA molecules was visualized using Cytoscape software [92].

### 4.7. Analysis of Previously Published B. napus RNA-Seq Data

Transcript data for silique, stem, leaf, flower, and root tissues as well as salinity (200 mM), ABA (25 μM), and cold (4C) stresses at 4 and 24 h after treatment and dehydration stress at 1 and 8 h after treatment are related to the study of Zhang et al. [47]. These data are available with the project ID CRA001775 at [93]. Initial quality analysis was performed on FastQ files using FastQC software [94], then preprocessing of raw sequence data was conducted and low quality reads, adapter sequences, and duplicate mapping reads were filtered using Trimmomatic on Linux [95]. The preprocessed FastQ files were aligned to the Brassica napus reference genome using STAR [96]. The counts obtained from STAR normalized to transcript per million (TPM). Log2 (TPM + 1) used to generate the heatmap utilizing TBtools [46]. Clustering the data was performed using the Pearson correlation coefficient and the complete linkage method.

### 4.8. Codon Usage Bias Analysis

The sequences were evaluated for the frequency of optimal codons (FOP), GC content, GC content at the third site position of codon (GC3s), codon adaptation index (CAI), effective codon number (ENC), and relative synonymous codon usage (RSCU) for the BnATGs using CodonW 1.4.2 [97]. Statistical analysis was performed using Excel software. Clustering the data was performed using the Pearson correlation coefficient and the complete linkage method using TBtools [46].

## 5. Conclusions

In recent years, bioinformatics tools have been used to identify important genes in increasing plant tolerance to biotic and abiotic stresses. On the other hand, autophagy-related genes have important roles in plant growth, development, and responding to environmental stresses. Therefore, in the present study, 127 BnATGs were detected using bioinformatics tools in rapeseed. The reason for expanding this gene family was the tandem and segmental duplications. Ka/Ks for most of the paired genes were <1, indicating a negative selection during the evolution of *B. napus* plants to maintain their function. Promoter analysis showed hormone- and stress-responsive elements in the *BnATGs* promoters, which is in line with gene ontology results suggesting their role in various plant biological processes. Likewise, the expression patterns of *BnATGs* ascertained their roles in different tissues under various environmental stresses in *B. napus*, which can be applied to develop stress-resistant cultivars in future studies. Besides, the mutation was the main factor in the formation of *BnATGs* codons. In addition, they are more likely to have A/T at the 3′ end of their codons. This study was performed to detect the molecular evolution and the possible function of *BnATGs*, and it has provided useful information about *BnATGs* for future studies.

## Figures and Tables

**Table 1:** Simple sequence repeats detected in *BnATGs.*

Gene ID	Count	Motif	Gene ID	Count	Motif
BnATG1a	2	(GGTT)3, (ATC)4	BnATG13a	2	(AAC)4, (TCC)4
BnATG1b	4	(ATTT)3, (TC)7, (GGA)4, (GGC)4	BnATG13b	1	(TCT)4
BnATG1c	1	(GATG)3	BnATG13d	2	(GAT)4, (TCT)5
BnATG1f	2	(GGTT)3, (ATC)4	BnATG14a	1	(AC)6
BnATG1g	1	(ATTT)3	BnATG14c	1	(AC)6
BnATG1i	2	(GATG)3, (TTTG)3	BnATG14e	1	(GGAAC)3
BnATG3d	1	(GAG)5	BnATG16a	1	(TGATT)3
BnATG4c	1	(GAAGA)3	BnATG16b	1	(TTTGA)6
BnATG4d	1	(TCTA)3	BnATG18a	3	(TTCC)4, (TCT)4, (GGA)5
BnATG5a	2	(CTTT)3, (CCT)5	BnATG18b	1	(GCA)4
BnATG5b	1	(AGA)7	BnATG18e	1	(CTC)4
BnATG5c	1	(TTTC)3	BnATG18g	1	(GGT)4
BnATG6a	3	(GAA)4, (TG)10, (GT)7	BnATG18h	1	(TTTTAT)3
BnATG6b	1	(GAA)4	BnATG18i	1	(CAG)5
BnATG6c	1	(GAA)4	BnATG18l	4	(GAT)4, (AGC)5, (ATG)4, (TTC)4
BnATG6d	1	(GAA)4	BnATG18m	2	(GAT)6, (TTC)4
BnATG7a	1	(TTTC)3	BnATG18r	1	(CTC)4
BnATG8c	2	(TTTG)3, (TTC)4	BnATG18x	3	(ATG)4, (CAT)4, (TTC)5
BnATG8d	2	(TATTT)3, (TTG)5	BnATG20a	2	(AAC)4, (TC)7
BnATG8h	2	(ATTCA)3, (GTT)4	BnATG20b	3	(ATA), (TC)7, (AAC)4
BnATG8j	2	(TCT)5, (AT)12	BnATG101b	1	(TCG)4
BnATG8k	1	(TTTGA)3	BnATG101c	5	(TGGCCT)3, (CTA)6, (TTTA)4, (GATG)3, (CCAT)3
BnATG8l	3	(AAGC)3, (TTGA)3, (TTCT)4	BnATG101d	3	(CCAT)3, (AT)7, (TTC)4
BnATG8o	1	(TA)8	BnATG101e	2	(TCT)4, (TC)6
BnATG8p	1	(CTT)4	BnTORa	5	(TTTA)3, (TC)7, (CT)8, (TC)8, (CTTT)3
BnATG8q	3	(TTTG)3, (TC)7, (TTC)4	BnTORb	3	(TCTT)3, (AT)9, (ATT)4
BnATG8r	1	(TTG)6	BnTORc	2	(TATTT)3, (TTA)5
BnATG8y	2	(AAGC)4, (AT)7	BnVPS15a	2	(TTTC)3, (TTTG)3
BnATG9a	7	(TCAAT)5, (CT)10, (CTC)4, (GAG)5, (GAT)5, (AAGA)3, (GTTA)3	BnVPS15b	2	(TTTC)3, (TTTG)3
BnATG9c	6	(TCAAT)4, (CT)10, (GAT)4, (TATT)3, (AAGA)3, (GTTA)3	BnVPS34a	1	(TA)7
BnATG10a	1	(CGGCAG)3	BnVPS34b	1	(TC)6
BnATG11a	2	(TTCT)3, (TTTA)5	BnVTI12a	1	(AAG)6
BnATG11b	3	(TTAT)3, (AGA)6, (AAC)4	BnVTI12b	3	(CCTT)3, (AAT)5, (TCA)4
BnATG11c	1	(ATC)4	BnVTI12d	1	(AAG)5
BnATG11e	1	(AT)6	BnVTI12e	1	(CCTT)4
BnATG11f	1	(AAC)7	BnVTI12f	2	(ATAC)3, (CTT)4
BnATG12a	1	(TTTGT)3			

## Supplementary Materials

The following are available online at https://www.mdpi.com/2223-7747/9/10/1393/s1, Table S1: Features of rapeseed ATG proteins, ATG1/13 kinase complex, PI3K complex, ATG9/2/18 complex, ubiquitin-like ATG8, and PE conjugation pathway, ubiquitin-like ATG12, and ATG5 conjugation pathway, and SNARE groups shown in yellow, green, gray, red, blue, and pink colors, respectively; Table S2: Ka/Ks analysis of the BnATG duplicated paired genes, positive selection, segmental, and tandem duplications shown in yellow, blue, and green colors, respectively; Table S3: ATG protein domain. The existence of ATGs domains in the Arabidopsis, *O. sativa*, *C*. *sinensis*, *N. tabaccum*, and *V. vinifera* sequences was assessed using HMMscan databases; Table S4: Gene annotations of BnATGs, biological process, cellular component, and molecular function are shown in yellow, brown, and blue colors, respectively; Table S5: List of cis-acting elements in BnATGs promoters, cell-cycle- and tissue-specific, hormone-responsive, light-responsive, regulatory, stress-responsive, and unknown function elements are shown in red, yellow, blue, brown, green, and gray colors, respectively; Table S6: Putative *BnATGs*-targeted miRNA; Table S7: RNA-seq data analysis of *BnATGs*, the color boxes indicate expression values, the lowest (blue), medium (Pale goldenrod), and the highest (red); Table S8: Relative synonymous codon usage analysis (RSCU) of BnATGs.
