# Supplementary material for: In Silico Analyses of Autophagy-Related Genes in Rapeseed (Brassica napus L.) under Different Abiotic Stresses and in Various Tissues
Source: Plants (Basel). 2020 Oct 20;9(10):1393. doi: 10.3390/plants9101393 (PMC7594038; doi:10.3390/plants9101393)
Supplement: Supplementary file 1 [file plants-09-01393-s001.pdf]

Responses to Reviewers

Dear Editor,

Please find enclosed our manuscript, which is the revised version of plants-929167, entitled “Genome-Wide Identification, Molecular Evolution, Codon Usage, and Expression Analyses of Autophagy-Related Genes in Rapeseed (*Brassica napus* L.) Under Different Abiotic Stresses and in Various Tissues” as contribution to the special issue “The Impacts of Abiotic Stresses on Plant Development”.

We highly appreciated all the constructive comments and paid heed to all of the suggestions which enrich this manuscript. We believe that the content and the clarity of our manuscript are improved in the revised version. Please note that the changed parts are highlighted in the revised manuscript.

We hope that you will enjoy reading this new version and find it suitable for publication in Plants.

Thank you by advance for applying a publication fee waiver if the paper is accepted, as agreed earlier with Justus Wu then Harry Fu.

Thank you for your consideration.

Dr. Cédric Jacquard

On behalf of the authors

Please find enclosed the responses to reviewers :

****

Reviewer #1:

****

This is an interesting manuscript because the authors characterize the autophagy-related genes of Brassica napus under abiotic stress. I agree with the authors when they state that ATGs are largely unknown in this species. They detect 127 BnATGs using bioinformatics tools and analyze the expression patterns under various tissues that provide useful information about this class of genes for future studies. Although I appreciate the work conducted in this study, I have some concerns that, in my opinion, deserve to be considered by the authors to gain further clarity and impact. Below I go through them in detail.

Author’s Response: We are thankful to the reviewer for positive comments to improve the manuscript.

1. All the supplementary tables should be more detailed. For example, a sheet explaining metadata,

color code, analysis, etc. could be added to each table and will be very useful for the community.

Author’s Response: Based on the opinion of the respected reviewer, the supplementary tables were grouped according to different colors and the explanation of colors for each table has been added to the caption of each table.

2. Line 136: What are the 165 paired genes measured? why isn't it 127? Please explained.

Author’s Response: Thank you for your very careful review of our paper. Based on the set criteria for identifying duplicated paired genes (more than 80% identification of the aligned region and more than 80% alignment coverage compared to the longer genes), it was determined that some genes have more than one homologous gene, thus, they participate in several duplications. For instance, the *BnATG18v* gene is homologous to the *BnATG18g*, *BnATG18e*, *BnATG18j*, and *BnATG18o*, and the paired genes including *BnATG18v/BnATG18g* ،*BnATG18v/BnATG18e*, *BnATG18v/BnATG18o*, and *BnATG18v/BnATG18j* have been considered as duplicated. Therefore, a total of 165 pairs of duplicated genes were identified and the selection pressure (Ka / Ks) was studied on them.

3. Figures do not appear when quoted and make reading very difficult.

Author’s Response: The figures are high-quality, but when they are placed in the manuscript (based on the journal format), their quality decreases, thus, separate files of images have been attached to the manuscript. Likewise, by increasing the scale of the images in the manuscript, the images are completely clear and legible.

4. Line 220-228: The list of SSRs could be detailed in a table to help understand.

Author’s Response: According to the reviewer's suggestion, the table of identified SSRs in *BnATG*s has been added to the MS and highlighted as a yellow color.

5. Line 231: The text does not explain the result of figure 9. Please, explain more the inference of the miRNA-gene network (methods, results …).

Author’s Response: Thank you for pointing this out. To avoid misunderstanding the reader, the SSR section has been separated from miRNA and you can read the descriptions related to the miRNA-gene network in the text in lines 234 to 240 in the result section and lines 393 to 411 in the discussion section.

6. figure 6 and 7 are very hard to understand. Why not display the same color code for the same

patterns shared by all the sequences? This will simplify the reading, limit the number of legends, and allow the authors to detail the different motifs (what is motif 1, 2…).

Author's Response: This analysis aimed to identify conserved motifs, the motif patterns in protein sequences, and finally to investigate the differences within the group. Accordingly, the results are specific to each ATG group. For example, in the ATG1 and ATG8 groups, twenty conserved motifs were identified and the colors of the identified motifs were same in the both groups, but these motifs were completely different in terms of sequence and motif distribution. For instance, motif 2 is highlighted in yellow while motif 2 sequence, location, and length are quite different in the ATG1 and ATG8 groups. On the other hand, the separate analysis of each group allows us to examine the differences within the group. As mentioned in the discussion of the phylogenetic tree, the identification of conserved motifs complements the results of phylogenetic studies. If the identification of conserved motifs of all ATG sequences was performed simultaneously, we were not able to establish a relationship between the detected motifs and the corresponding phylogenetic tree.

Likewise, due to the number of groups as well as the identified motifs in each group, it is virtually impossible to provide a sequence related to each motif or logo in the results. Many motifs are also non-functional and only indicate the amount of conservation and the difference between the members of each group. It should be noted that in the supplementary table S3, specific and functional domains are presented for the genes of each group.

7. My major concern is the materials and methods section that requires some improvement. I understand that all protocols of RNAseq have already been used and published (Zhang, Y., Ali, U., Zhang, G. et al., 2019), but the literature is useful to get into deeper detail if needed, but not for the basics. I suggest including a brief description of the major steps (those important to make sure that all quality standards were met) for all methodologies described in this manuscript. Please note that to review this section of the manuscript in detail. The authors need to detail more the experimental conditions and especially the RNA-seq analysis under which these data were produced. Because of these forgetting, the transcriptomics results of section 2.7 and 2.8 are difficult to exploit and verify.

Author's Response: Thank you for your helpful comments. According to these suggestions, more details related to the RNA-seq data (based on the paper of Zhang et al. 2019) have been added to the MS and highlighted as a yellow color.

8. figure 10 has upper case letters A and B but the nomenclature in the text is requested in lower case.

Author’s Response: Thank you for catching this mistake. It has been corrected and highlighted as yellow color in the MS.

9. Still on Figure 10 and the transcriptomics results, the authors should use a cluster classification of their data to highlight tissue-specific and abiotic stress profiles.

Author’s Response: Based on the reviewer's suggestion, a cluster classification has been added to fig. 10 for a better understanding by readers.

10. Authors should also use a cluster classification of their data in Figure 11 to better demonstrate codon specificity or of each BnATGs.

Author’s Response: Based on the reviewer's suggestion, a cluster classification has been added to fig. 11 for a better understanding by readers.

****

Reviewer #2:

****

My main concern with this paper is that it is simply presenting the results of bioinformatics programs applied to published data. As a small portion of a scientific paper, this is fine. But I would expect that the authors' bioinformatics findings' would be i) tied to a theoretical context that was developed, tested, and reported on by the authors as the main contribution of the paper and/or ii) used to justify additional experimental trials that the authors themselves have conducted and are reporting on as the main contribution of the paper. In these respects, the paper lacks a "research design", i.e., hypotheses being tested. Hence, there are no conclusions (of tested hypotheses) other than what the bioinformatics programs generated.

Author’s Response: We are pretty sure that the reviewer’s suggestion is valuable and will surely strengthen our manuscript, however, plant genome sequencing and development of algorithms, software, and databases of genomes, transcripts, etc. led to the use of bioinformatics analysis and database information as the first step in plant biology studies with different objectives. The study of gene families, which is widely used today for various plant genes, is one of the fields of plant biology that aims to identify, introduce, assess the evolutionary pathway, and determine the function of genes in the plant biological cycle. The purpose of these studies is to provide basic information for the correct selection of the appropriate genes to design future experiments. Similar to other gene families studies

(following references), the present analyses conducted on *BnATG* genes are divided into two sections: 1) analysis related to the evolution of *BnATG* genes and 2) analysis of the function of these genes using bioinformatics methods. Phylogenetic relationships, duplication, selection pressure, gene structure, intron splicing, and codon usage were evaluated in the section related to the evolutionary analysis. The mentioned Analyses are based on *in silico* methods. Prediction of *BnATG*s function, including subcellular localization, identification of cis-elements in the promoter region, identification of miRNAs affecting post-transcriptional regulation, and evaluation of gene expression based on RNA-seq data available in databases. We are pretty sure that the respected reviewer's suggestion is valuable and will surely strengthen our manuscript, however, 127 *BnATG* genes have been identified in this study and it is certainly not possible to laboratory investigate the mentioned functional analyses in all of them. On the other hand, due to the Covid-19 pandemic, the authors cannot conduct experimental studies on the function of some *BnATG* genes that complement the results of bioinformatics analyzes and this part of the study will be conducted at the right time. Another important part of gene family analysis is to investigate their expression under different stresses, tissues, and developmental stages of the targeted plant. Although in the section of the *BnATG* expression profile, the data from the study of Zhang et al. (2019) has been used, bioinformatics analysis and determining the expression level of rapeseed genes and then isolating the expression data of *BnATG* genes in tissues and stresses were carried out in the current study. Likewise, according to the number of biological replications (74 samples with three biological replications for stresses and two biological replications for tissues), the presented data of the *BnATG* gene expression is accurate. Finally, some of the similar papers published in 2020 are listed as follow:

Sharma, B., & Taganna, J. (2020). Genome-wide analysis of the U-box E3 ubiquitin ligase enzyme gene family in tomato. Scientific Reports, 10(1), 1-15.

Zhang, Y. M., Chen, M., Sun, L., Wang, Y., Yin, J., Liu, J., & Hang, Y. Y. (2020). Genome-Wide Identification and Evolutionary Analysis of *NBS-LRR* Genes From Dioscorea rotundata. Frontiers in Genetics, 11, 484.

Wang, Y., Zhao, Y., Yan, M., Zhao, H., Zhang, X., & Yuan, Z. (2020). Genome-Wide Identification and Expression Analysis of TALE Gene Family in Pomegranate (*Punica granatum* L.). Agronomy, 10(6), 829.

Ren, Y., Ge, D., Dong, J., Guo, L., & Yuan, Z. (2020). Genome-Wide Identification and Expression Analysis of MAPK and MAPKK Gene Family in Pomegranate (*Punica Granatum* L.). Agronomy, 10(7), 1015.

Zhu, W., Wu, D., Jiang, L., & Ye, L. (2020). Genome-wide identification and characterization of SnRK family genes in *Brassica napus*. BMC Plant Biology, 20, 287.

Zhao, Y., Zhao, H., Wang, Y., Zhang, X., Zhao, X., & Yuan, Z. (2020). Genome-Wide Identification and Expression

Analysis of *MIKC-Type MADS-Box* Gene Family in Punica granatum L. Agronomy, 10(8), 1197.

Rehman, A., Atif, R. M., Qyyum, A., Du, X., Hinze, L., & Azhar, M. T. (2020). Genome-wide identification and characterization of *HSP70* gene family in four species of cotton. Genomics. 112 (6): 4442-4453.

Wang, T., Hu, J., Ma, X., Li, C., Yang, Q., Feng, S., ... & Song, X. (2020). Identification, evolution and expression analyses of whole genome-wide TLP gene family in *Brassica napus*. BMC Genomics, 21(1), 1-14.
